# Domestic migration and mobile phones: A qualitative case study focused on recent migrants to Ouagadougou, Burkina Faso

**DOI:** 10.1371/journal.pone.0236248

**Published:** 2020-08-06

**Authors:** Jude Mikal, Kathryn Grace, Jack DeWaard, Molly Brown, Gabriel Sangli

**Affiliations:** 1 Department of Health Policy and Management, School of Public Health, University of Minnesota, Minneapolis, MN, United States of America; 2 Department of Geography, Environment and Society, Minnesota Population Center, University of Minnesota, Minneapolis, MN, United States of America; 3 Department of Sociology, Minnesota Population Center, University of Minnesota, Minneapolis, MN, United States of America; 4 Department of Geographical Sciences, University of Maryland, College Park, MD, United States of America; 5 Institut Supérieur des Sciences de la Population, University Joseph KI-ZERBO, Ouagadougou, Burkina Faso; Sam Houston State University, UNITED STATES

## Abstract

Migration has long been used as a strategy for livelihood diversification in rural, subsistence communities. Yet in order for migration to effectively serve as a livelihood diversification strategy, it should meet certain conditions: migration should ease financial burdens, should confer access to economically valuable resources and information, and should broaden social networks. Using qualitative data gathered in 25 interviews with rural migrants to Ouagadougou, Burkina Faso, we examine how mobile phone technology has impacted migration as a livelihood diversification strategy. Our results show that while mobile phones facilitate migration, the advantages conferred may benefit migrants at the expense of the home communities. Mobile phones alleviate financial constraints, enable access to broader networks, and facilitate informational and resource support ***among migrants***. Our results show limited evidence of migrants using mobile phone technology to provide resources or information to the home community. Our results highlight the need to reconsider the ways in which migration can be used as a livelihood diversification strategy in light of changing communication technologies to promote the economic success of both migrants and their home communities.

## Introduction

Given the increasingly variable climates, extensive poverty, limited infrastructure and dearth of economic opportunities, perhaps no other families on Earth are as vulnerable to livelihood instability as subsistence farming families and communities in West Africa. One potential strategy for mitigating household livelihood insecurity is out-migration–where select individuals from farming communities leave their origin community in search of other opportunities to earn money. This is part of a well-documented “livelihood diversification” strategy widely adopted in West Africa [1–5]. In rural West Africa, these moves are very often short-term with the migrant eventually returning home to the community of origin and are part of a broader and well-established pattern of circular migration (coming and going from the origin community as needed or according to the community’s labor demands).

Migration may diversify livelihoods by providing the opportunity to gather information in new communities to be transmitted back to the origin community. It can also provide access to remittances and other economic resources, expand and proliferate valuable social ties, and ease resource limitations (in other words, fewer mouths to feed) facing the family of origin (see [6–8]. Each of these aspects of ***livelihood diversification*** may be facilitated by the rapid expansion of mobile phone technology, which, even in rural West Africa, facilitates low-cost and frequent interaction with friends and family [9–13]. However, literature from migration studies has been inconclusive, showing that technology can both facilitate and hinder migration. Similarly, literature on development and mobile technology indicates that while mobile technology may serve as a low-cost way to spread information, it may also reinforce gender and socioeconomic divides [14,15].

In this research we use qualitative focus groups to investigate the ways that out-migrants from rainfed dependent agricultural communities use mobile phones to interact with and provide information to their origin communities. This research was driven by an interest in understanding how urban migrants from rural communities can buffer against the economic stresses of food insecure regions by providing remittances and information, diversifying social networks, and alleviating economic stresses on the family. It was also driven by an interest in better understanding how mobile technologies are used by migrants. We are specifically interested in examining the following questions: How are mobile phones used to promote information, resource, and other support exchange between migrants and their origin communities? How does mobile phone use impact relationships between migrants and home communities?

Data collection for the project was completed in the summer and fall of 2016 in peri-urban and urban areas of Ouagadougou, Burkina Faso in a series of four focus groups with five participants in each, followed by five individual interviews (total sample size is 25). The interviews explored how individuals from poor subsistence communities used mobile phones throughout the migration process in an effort to better understand how mobile phones facilitate connections and resource exchanges (resources of any type, including information, support, or economic) between migrants and their communities of origin. Results from the focus groups are useful for better understanding the ways that urban and rural communities connect via mobile phones and in describing how individuals from rural communities use their mobile phones to adjust to new living conditions and lifestyles in urban areas while still engaging with families, friends, and neighbors from their home communities. The information also contributes to expanding scientific understanding of the complexities underlying migration decisions, interactions between origin and destination communities, and the integration challenges that migrants face.

### Background

Migration is an adaptive strategy used to mitigate the destabilizing impacts of climatic or weather variation on livelihoods [4,5,16]. The key idea is that households attempt to diversify their livelihood portfolios by sending one or several members out as migrants [3,17,18]. These migrations capitalize on employment and wage opportunities in other places. The resulting financial resources are then remitted and/or taken directly home and used, for example, to diversify agricultural production (invest drought-resistant crops, irrigation equipment, etc.), as well as for other production- and consumption-oriented activities [19,20].

Recent technology development, including mobile phones and airtime through low-price phone cards, play a potentially important role in rural to urban migration [21–25]. By providing near-continuous access to networks of support in the home communities, technology provides a space-time compression: enabling quick and easy communication over large distances, transforming long-distance communication from an intermittent to a near-daily occurrence. Doing so has a dually facilitative effect on migration. First, daily interaction with home communities reduces the costs of migration by contributing to the maintenance of family and community ties, enabling the coordination of household activities from afar, and facilitating not only the communication of remittances—but the management, coordination, and distribution of those remittances. Second, communication technology can have a catalyzing effect on “chain” migration by providing information on migration destinations [26]. For example, in one study of migration from Iraq and the Sudan, Mikal and Woodfield [27] suggest that technology can also ease the stress of migration upon arrival by promoting social integration and reducing time costs associated with daily hassles.

In addition to a focus on the facilitative effects of technology in migration, research on migration and technology has also tended to group information communication technologies into a single category that includes both Internet communiqué and mobile technology. Nevertheless, research on the ways in which technology impacts movement within and between the Global South might do better to focus uniquely on the role of mobile phone technology, given not only lower rates of formal education and a preponderance of illiteracy (of which Burkina Faso has one of the highest rates in the world)—but also the cultural value assigned to both social networks and oral traditions. In fact, Vertovec [28] and De Bruijn [29] suggest that perhaps nothing has served to connect more individuals across time and space than the widespread adoption of new and inexpensive telephone technologies, referring to new communication technologies as the new glue binding communities together across large geographic distances.

With respect to the ***impact*** of communication technology on the likelihood of migration and the migration experience, the preponderance of empirical work has focused on the facilitative aspects of connectivity [30]. In the case of climate-migrants, Boas [10,11] suggests that communication technology has the potential to expand and proliferate social networks while also providing bi-directional information exchange between the migrants and their community of origin–and that the geographic dispersion of ties can help to increase the likelihood of migration. In addition, several studies have pointed to the value of technology and mobile phones in the transmission of economic resources, though principally via remittances, with less emphasis on resource transfers ***to the migrant*** from the home community [12,30].

Despite immense potential for mobile phone technology to mobilize populations, more recent research has begun to examine the drawbacks of communication technologies. Conversations with what Hannaford [31] characterizes as “remittance hungry relatives (p. 47),” or the burden of problems at home, can exacerbate stress for migrants. In addition, several authors have noted the unwelcome influence of migrants in both household management and surveillance that is afforded by mobile phones (and associated technologies). Hannaford [31] interviewed 54 Senegalese women married to or divorced from Senegalese migrants and recounts the difficulties associated with the continued dominion over both household and time management exercised by their stay-at-home partners through the use of mobile phones, with some men using remittances to relatives to secure control over their partners. This continued communication through mobile phones has even been shown to inhibit return migration. In a multi-site ethnographic study, [24] tells the story of West and North African migrants living in worker hostels in France, and shows the role played by mobile phones in their continued cultural connection and the decision to return home following retirement. According to the study, mobile technology can discourage return migration by both promoting connections within the host culture and by increasing pressures to provide resource support for families and communities back home.

### Theoretical models and lingering questions

**Fig 1:**
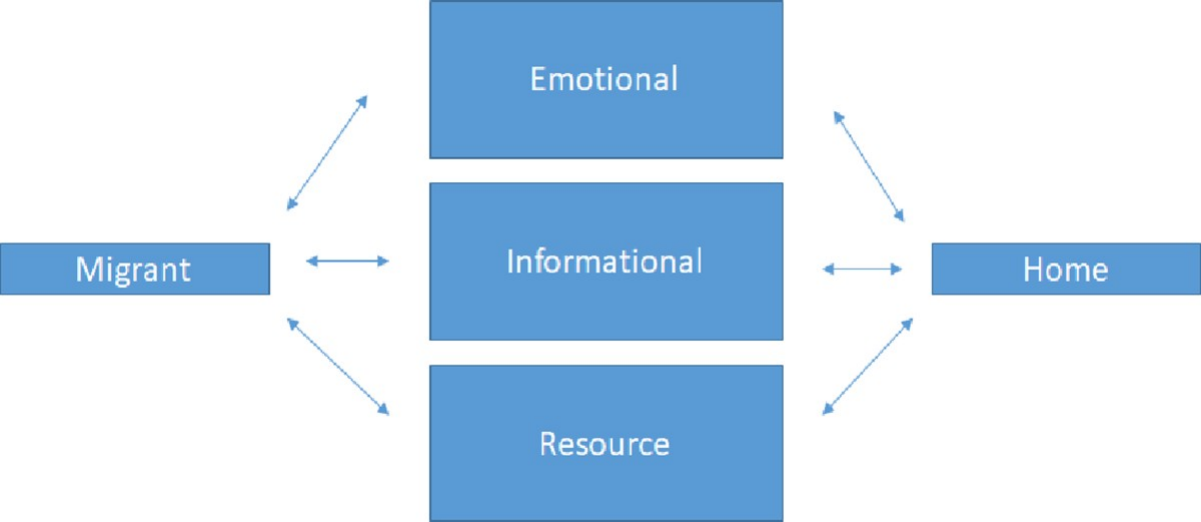
Theoretical model of support between migrants and their communities/households of origin. Arrows indicate that migrants and members of the communities of origin both may receive and offer different types of support.

In the fields of social psychology and sociology, support is often divided into three categories: informational, instrumental, or socio-emotional. ***Informational support*** refers to the exchange of information related to a particular problem or stressor, while ***instrumental support*** refers to the exchange of goods or services related to a problem and can include money, resources or time. Finally, ***socio-emotional support*** refers to emotional support transmitted through a feeling of togetherness or a feeling that one is esteemed by friends and family (see e.g., [32]. While these mechanisms for support transmission have most frequently been used to describe stress-reduction through social interaction, they map onto the needs of migrants and can be useful in helping researchers to identify key questions around mobile phones and migration. Specifically, if support can be transferred in the form of socio-emotional support, informational support, and instrumental support, and can be transferred both from the host community to the migrant, there are six support transmission options as shown in Fig 1, below.

This theoretical model helps us to identify four opportunities for additional research on mobile phones and migration. First, while research on migrants has not typically been subdivided by the nature and quality of support exchanged, research has focused on resource transmission from the migrants to the home culture through remittances and on the role of home cultures in providing emotional and informational support to the migrant (see for example, [33]. What then is the role of the home communities in providing resource support ***to*** migrants, or how migrants can be called upon to ***provide*** informational or emotional support to home communities. Second, while Perkins and Nuemayer [26] and others have shown that information provided by previous migrants can serve to catalyze chain migration, we know very little about the emotional and resource support provided by in situ migrants. Third, while research has focused on international migration, where face-to-face communication is often not possible, very little research has discussed the role of mobile phones in domestic migration, where face-to-face communication is still possible. Specifically, when support transmission is still accessible in person, what types of support are most easily transferred to online spaces? And finally, what are the disadvantages of information and communication technologies (ICTs) in domestic migrations?

The degree to which migrants provide access to economic resources and economically valuable information, continue to connect and engage socially with home communities, and continue to constitute a financial burden on communities of origin have important implications on the degree to which migration can be viewed as a profitable livelihood diversification strategy. Mobile phones have transformed the degree of engagement between migrants and their home communities in ways that have the potential to be both supportive and burdensome. In this paper, we examine bi-directional transfers of support to better understand the benefits ***and the drawbacks*** of mobile phone use during migration and how such continued engagement impacts migration as a livelihood diversification experience.

### Setting

The setting for our investigation of the benefits and drawbacks of mobile phone use is Burkina Faso. Burkina Faso is one of the poorest countries in the world. It is ranked 46^th^ on the 2019 Human Development Index of African countries, and 182^nd^ out of 189 countries in human development, according to the UNDP’s 2019 Global Country Rankings report. The majority of the population is dependent on small-scale farming for food and income [34]. Educational attainment is low and nearly 60% of the total population are not functionally literate [35]. Like many sub-Saharan African countries, Burkina Faso has experienced substantial urban growth over the past 50 years, with the much of the urban population growth having occurred in the capital city of Ouagadougou [36]. Compared to local populations, migrants are more often unemployed, self-employed or employed in informal labor markets, with female migrants more often employed in informal markets [37]. More recently, mobile phone usage has rapidly increased in Burkina Faso [38]. As in other regions of sub-Saharan Africa, mobile phones are more often owned by men and by those with more education and those living in urban areas [15,38,39]. However, research on gender and mobile phone usage indicates that in Burkina Faso women who own mobile phones may have better access to healthcare than women without phones [38].

## Methods

The University of Minnesota, Twin Cities Institutional Review Board approved this project on 5/17/2017 (1605S87803). Consent was obtained orally from all study participants.

Our aim was to explore the technology-mediated transfer of advice, information, and resources between migrants and their communities of origin with the idea that recent migrants to urban areas have access to knowledge that might support farmers in their home communities. We explored this topic in a series of focus group interviews with rural-to-urban migrants living in Ouagadougou, Burkina Faso. We selected qualitative research methods for our research design given that the purpose of the research was to uncover not just the relationship between technology and migration, but also why migrants use technology and how technology influences the process of migration, from the migrant’s standpoint. These questions of “how” and “why” lend themselves to a qualitative research design in which participants describe their perceptions and experiences in their own language in order to ensure that observed patterns and relationships are situated in the culture and context in which they are observed. We use focus groups as they offer a number of advantages over one-on-one interviews. Specifically, focus groups are conducive to exploratory research as they serve to de-emphasize the role of the researcher in the interview process and to highlight interactions between the participants, themselves. As such, respondents and researchers are able to immediately triangulate observations for validity [40–42]. Conversely, focus groups can be logistically difficult to organize, prone to overrepresenting perspectives of talkative or domineering participants, and participants may find sharing sensitive or emotional information embarrassing or off-putting. Used in developing world contexts, there is also evidence to suggest that language differences along with power differentials between interviewers and participants, or between participants themselves, may hinder effective communication [43].

To mitigate some of the disadvantages associated with focus group interviews, we began by ensuring linguistic and cultural concordance between our interviewer and research participants, and between participants themselves. All focus groups were conducted by a local collaborator from the University of Ouagadougou, and overseen by authors GS, a professor of Demography at the University of Ouagadougou and JM, from the University of Minnesota, Twin Cities School of Public Health. In the days leading up to the interviews, site visits were organized in neighborhoods around Ouagadougou known to have large migrant populations. Pre-interview site visits were structured around making contacts with local residents, often at cafes and other establishments. Researchers would introduce themselves to merchants and other business owners, and ask for referrals to migrants, especially seasonal migrants, living in the area. Sometimes the merchants themselves were migrants, and, if not, most merchants were able to make a referral—and the remainder of the group was recruited using a snowball sampling technique [44]. Because of this, many of the migrants hailed from the same home community and knew each other socially prior to participation in the focus group interview. Migrants were offered 1,000 Central African Francs ($1.65) as compensation for their time and any transportation costs associated with getting to focus groups.

The focus group interviews took place in July and August of 2016 in neighborhoods in and immediately around Ouagadougou. Focus groups were conducted by our local research collaborator in Mooré (the most common local language). Each interview lasted approximately two hours and followed a semi-structured protocol. Four main themes were introduced: (1) motivations for migration and the decision to leave the village; (2) bilateral transfers of support and capital to facilitate migration; (3) mobile phones and use as facilitators of migration; and (4) mobile phones and use as barriers to migration. In order to address concerns over the sensitivity of information shared regarding stresses and setbacks associated with migration, we used a strategy called *indirect* or *third party proxy* questions [45]. This means we asked participants to tell us about the migration experiences of a close friend or family member. This is intended to allow for the free exchange of more personal or sensitive information without necessarily disclosing a participant’s own experiences, and is a common technique used to mitigate concerns regarding self-disclosure.

Focus groups were audio-recorded and translated and transcribed by Mooré / French bilingual research assistants at the University of Ouagadougou. The transcripts were then reviewed by our collaborator who had collected the focus group data to ensure clarity and accuracy. Author JM met regularly throughout the data collection process with our local collaborator to discuss emergent themes, and each participated in the initial coding using a constant comparison approach to allow themes to emerge directly from the data [46,47]. Author JM then reviewed the transcripts again using a top down coding technique to find illustrative quotes from the focus group interviews. All quotes used in the manuscript were translated from French to English by author JM, and reviewed by our local collaborator and KG to ensure accuracy.

### Participants

Four focus groups were conducted with five participants each. A fifth focus group was scheduled with migrant farm workers at the Barrage de Boulmiougou, but agricultural producers in that area preferred not to leave farms unattended and thus sat for a series of 30–45-minute individual interviews. As such, the final five interviews were conducted in succession. As is typical for focus group interviews in developing countries, men and women were interviewed separately. This convention in focus group interviewing is important for a number of reasons, most notably when there is a perceived power differential between men and women, when men and women occupy different social roles, or when answers to interview questions are likely to differ between men and women (see e.g., [48–51].

To determine an appropriate sample size, we looked for evidence of thematic saturation–or a point at which answers to our research questions were met with the same or very similar responses [52]. Research on focus groups suggests that at least 80–90% saturation occurs within three to five focus groups [53]. In our sample, general theoretical saturation emerged following the individual interviews at the Barrage de Boulmiougou. In total, recruitment yielded 25 participants who participated in interviews.

Most participants were unskilled laborers, working in a range of jobs including sales, construction, security, or food service. Participants had little to no formal education, with most reporting no formal education. However, the women migrants generally reported a higher level of education than the men. Participants ranged in age from around 23 years old to nearly 40, with most participants in their late-20s or early 30s. Most of the male migrants were married with at least one child—only two participants had four children and no participants had more than four. Only three male participants were unmarried with no partner, and all male participants with children reported being married. Women’s family situations were often more complex, characterized by either divorce or widowhood, children with more than one partner, and children who had to be left behind with family members in their origin communities.

Participants are identified using letters to represent their names. We chose this approach to maintain confidentiality. Culturally relevant names were not used because of the religious and cultural significance of names in Ouagadougou (for example, an individual may have two first names depending on the religious make-up of his/her family) which made selecting pseudonyms potentially problematic for the purposes of this project.

## Results

In this section, we use qualitative evidence to explore the interactions and transfer of resources between migrants and their home communities, and to identify the impact of these transfers on the migration process. Results are divided into the four principal categories outlined in our **Theoretical Models and Lingering Questions** section, above: (1) the exchange of advice, information, and resources between migrants and their sending communities via mobile phone, (2) technology use and capital exchanges between migrants, (3) ICTs and mobile telephony as catalysts and facilitators of domestic migration, and (4) drawbacks of ICTs and negative impacts on migration. Our results provide novel insights and promising new evidence of a multi-faceted use of mobile phones and voice calls in domestic migration. Nevertheless, the results highlight untapped potential for new ways of using technology that can benefit both migrants and origin communities: specifically, our finding that origin communities may pester migrants for remittances and resources, and fail to transmit on important information that could benefit the origin community. This finding is discussed in more detail below.

### Emotional, informational, and resource support

#### Migrant support to communities

Previous research has examined support transfers and remittances in some settings and similar to related research findings, our results indicate that there may be gender differences in how mobile phones are used to transmit resources [14,33,54] For example, the men in our study seldom referred to remittances transmitted through mobile phones such as AirTel Money. Most often, men who sent resources back to origin communities preferred to carry money back personally and make public displays of generosity. Participants visited home communities only when they could afford both the travel costs and gifts for family, friends, village children, and even elders. According to Participant A,

It’s not like it was before. Just going home—20 km away—is going to cost you around 3,000 francs on motorcycle. Then you have to buy little things…bread, soda…to give out once you get there. Then if you see your sister or your aunt, you want to make sure you have 1,000 francs to give them…and if it’s to go see an elder you have to give them something too, even if it’s only 200 francs. (Participant A, male, focus group 1)

Strong community ties and broader notions of family may make return home particularly difficult, as migrants are expected to provide gifts to a large number of people within the home community. According to Participant A and other male migrants, the inability to provide these types of gifts can result in feelings of shame. As a result, several focus group participants reported either not traveling home to avoid shaming their family or a loss in status among community members. Others traveled back to the village at night so as not to draw attention to their homecoming.

For female migrants in our study, mobile phones enabled home communities to ***request*** resources, especially in service of the health and well-being needs of children left behind. For example, Participant P migrated to urban Ouagadougou to keep house for her grandmother before beginning an internship to learn to braid and style hair. During her training, she began dating a man and subsequently became pregnant. Abandoned by the father of her child after he found out about the pregnancy, the participant entrusted the care of her daughter to her older sister back in the village, but continued to support the child financially and check in daily with her sister regarding her child’s well-being via mobile phone. Note that abandonment by male partners following a pregnancy can result in social ostracization for women, and often requires that young women leave the home community to save parents from the shame of a woman’s out-of-union pregnancy and leaving school. When asked if mobile phones have facilitated remaining in contact with her family in the village, the participant and author AY have the following exchange (note that *tô is a* common traditional meal in Burkina Faso prepared with corn or millet flour and accompanied by a sauce),

P: I don’t go a day without calling my son.

AY:…and how does it make you feel, to call home?

P: I called earlier and they told me he was sick, so I was able to send money. Since my son’s spent so much time here [Ouagadougou] he’s not used to food in the village and tô can be so heavy. So since he can’t eat tô, I send money. (Participant P, female, focus group 4)

Mobile phones facilitated the communication of resource needs, or financial obligation, on the part of those (women) entrusted with the care of female migrants’ children and this participant’s story of resource need communication and ICT-facilitated financial transfers was reported by other women interviewees. Mobile phones facilitating “care-taking” by women of other family members is one example of how mobile phone use may be gendered and has been documented in other settings as well (see [14].

Regarding information transfers, our results showed evidence that several of our respondents seldom provided information to their home communities; this included information related to either their personal lives and financial situation, but also included tips on available aid, agriculture prices, or economic opportunities. While migrants acknowledged that it would be possible for them to ask for financial assistance from friends and family in their home community, most were reluctant to disclose their circumstances for fear of losing status or creating worry. One participant from focus group one explains,

[As a migrant], you’re strong. You can work. You have problems here in the city, but you can’t go running to the village to tell them what’s going on—so you force yourself to keep going. You have your health, so you keep working. You lie or you don’t tell people at home what’s going on. It’s just you and God who know. (Participant A, male, focus group 1)

As a result, several respondents reported feeling trapped in Ouagadougou, forced to chase employment opportunities and skirt calls from friends and relatives in the home community who were awaiting their share of the fortune they assumed male migrants had undoubtedly accrued in the city. Additionally, our evidence revealed that migrants were unlikely to share economic information with their home communities. When asked directly whether they provided information on aid distribution centers or prices in the city, most migrants simply replied no.

This failure to communicate information from the city back to the village has several implications. First, it shows that there are still untapped opportunities in the use of mobile telephony in rural-to-urban migration in this West African context. Our results show that the pressure to provide resources when in contact with origin communities may make migrants reluctant to initiate any contact with family and friends, thus hindering communication that would facilitate the transfer of important information that could benefit members of origin communities, such as prices or aid distribution centers. In other words, fatigued by the harassment for resource support, several migrants reported simply cutting off communication with home communities. That said, migrants and their communities must also be able to imagine how such information could be useful. It is of no value to know the prices of tomatoes in Ouagadougou if the drivers who transport those tomatoes only offer to pay a flat rate.

#### Community support to migrants

With migration lasting anywhere from several weeks to decades-long relocations, mobile phones facilitated frequent communication that served to shore up family ties, allowing migrants to stay current on important news and events from the village and to attend to important cultural obligations. For example, while a number of respondents reported frequent calls home to ask about the health and well-being of parents and other family and community members, female migration frequently involved separation from one or more children—left in the care of a parent, or close relative in the village. This represented not only a financial hardship for the family entrusted with caring for the child, but also an emotional burden for the mother. When asked about calling home, participant Q reported, “*Why*, *just this morning I called to ask about my daughter—since she’s been sick*.*”* And when she asks how the calls make her feel, she replied,

Happy, of course! I gossip with my mom, and I can ask about my sisters and my children. Before, if I wanted news from home—someone had to leave the village and come all the way here, but now it’s easy! If you have 125 CFA, you can just call. (Participant Q, female, focus group 4)

Unlike transnational migrations, telephone technology was not reported to make communication of emotional and informational support possible, but rather lowered communication costs, increasing the frequency of support communication, perhaps decreasing the stresses associated with migrations and family separations.

Low-cost communication across large geographic distance, and the ability to connect with migrants irrespective of time and space, also enabled migrants to attend to important family obligations. Not only did ICTs facilitate the communication of resources in the situation described above, where mothers were separated from the daily care of their children, but migrants were also able to attend to other important obligations in the village, as well. Some male migrants described situations in which there were conflicts in the village, saying that through the use of mobile telephones they were able to participate in conversations and to offer perspectives and resources to help in problem resolution. One commonly recurring theme, mentioned in all four group interviews. was the issue of health and death, particularly as it relates to the death of a parent back in the village. Participant H illustrates the transition from no phone access to modern phone access,

I can tell you that—before, when there were funerals, we were sent out on our bikes to let everyone know. For example, we’d be in Benengo and someone would tell us, “Go tell everyone that one of the elders has passed away.” Now, we just tell everyone over the phone. Even when someone dies here in Ouaga, you just call. So that helps. (Participant H, male, focus group 2)

And it was in this manner that Participant T was informed of the death of her father when she was away from the village,

My dad died in Koudougou while I was away and I would never have known. But thanks to mobile phones, someone called from the village and all of my siblings were able to come to the funeral. Without a phone, someone would have had to send a letter—and you would never know if it came or not. (Participant T, female, focus group 4)

That older mechanisms of communication were not always reliable is a common theme in the transmission of both resources and information. The advent and widespread adoption of mobile telephones did not necessarily *create the possibility* of communicating emotional and informational support, but as a way of augmenting the reliability of communication networks, especially where more traditional transfers were seen as unreliable.

More limited research has examined the transfer of economic or resource support under the theme of support transfers ***from the home community to the migrant***. While existing research has examined the role of financial remittances from the migrant to the home community, the role of economic support *to migrants* is less clear, though research on transnational migration and migration systems has highlighted the dynamic two-way exchanges (including financial) between those in origin and destination areas [55–58]. Our results suggest that economic support from origin community family and friends plays a role in migrant success. In discussing remittance transfers, our interviewer asks whether participants have ever ***received*** financial support from family back in the village. One respondent responds, *“*

Yes! Often when things aren’t going well, I call and ask my family to go out and find money to send. My family back home knows that we came in part to relieve their burden…that it’s often harder for us here, than it is for them back in the village. (Participant A, male, focus group 1)

Participant A characterizes family back in rural villages as often “better off” than migrants to the city, and this characterization was not uncommon among respondents. Despite this, resource transfers from the home community to the migrant are seldom reported in literature on transnational migrations. This could indicate that mobile phones serve a different, bi-directional function in resource transfers in the context of domestic migration. In other words, while Central African Francs (CFA) have limited value in developed nations, money earned from planting and selling tomatoes, onions and okra may be enough to provide important financial assistance to domestic migrants. As a result, using mobile phones, migrants could communicate financial needs to their families back home and perhaps mobilize economic resources to serve as a financial safety net. Money can easily be sent to the migrant using the mobile phone via organizations like Western Union or AirTel Money.

### Valuable mobile phone exchanges among migrants

Our results also showed that migrants used mobile phones to communicate with other migrants. This strategy allowed migrants to leverage the knowledge, networks, and experience of other migrants and to pool commonly available resources. The transmission of advice, information and resources could be particularly valuable for migrants in search of employment and allow migrants to circumnavigate many potential pitfalls associated with employment seeking in the city.

Based on our interview transcripts, the reality for many migrants is that local employment opportunities in the city of Ouagadougou are locked up in well-established local networks and that employment opportunities are transmitted through those local networks via word of mouth. To access those networks, migrants place a special emphasis on “becoming native”—a process by which they make connections and inroads into a community, form relationships, and communicate both their search for work and the type of work they do. Until those relationships are established, migrants must travel for work, with one respondent reporting having biked from Ouagadougou to Bobo-Dioulasso (~400 km) to find work. This mobility has the dual impact of increasing the costs of looking for work and undermining local relationships by keeping migrants from implanting in a particular community and “becoming native” by keeping them on the move.

In addition, migrants report that they are subject to additional challenges associated with finding work. For example, migrants from the first two focus groups reported a process of “skimming” whereby a potential employer would communicate to a middleman that he was looking for a particular number of employees at 10,000 francs ($16.50) per day. The middleman would then offer those jobs to employees at a reduced rate, keeping the difference in price for himself. Despite this “skimming”, several migrants reported competitive under-bidding between migrants: jobs would be offered at a particular amount, but ultimately paid much lower as middlemen were incentivized to take the lowest bid possible and to keep the differential for themselves. In addition, even when jobs were taken and completed, there was a systematic abuse of migrant manual laborers through a pattern of non-payment or the imposition of unreasonable conditions for the payment of subsequent installments.

While mobile phones did not permit migrants to escape all of the pitfalls associated with precarious employment conditions, mobile phones did enable migrants to reduce some of the costs associated with looking for work. According to Participant O,

Without mobile phones, even finding work is hard. Nobody is going to walk around looking for you to come work. But with your phone number, it’s easy! (Participant O, male, focus group 3)

Participant U echoes a similar sentiment saying,

Phones are good. For example, if someone has my phone number and wants to give me work to support my family—he’s going to call me and say, ‘Hey! Where are you? Come to here or here, there’s work!’ That way he doesn’t have to come all the way to you. It’s faster—and you can go where the work is. Plus, when he calls you, you already know there’s work there (Participant U, male, Boulmiougou interviews).

He continues by saying that before there were phones, you might follow someone in the hopes of finding work, only to find that there was no work available and you would have wasted valuable time and money on transportation across the city. As such, mobile phones can help to reduce the costs of finding work. In addition, technology can potentially increase the value of migrant networks by allowing them to broker information on economic opportunity.

This brokering of economic opportunity can also be seen in other mobile phone communication among migrants. In our final set of interviews, we spoke primarily with migrants who were involved in gardening—principally growing produce such as tomatoes and strawberries. And while resource exchange still took place in person, resources and information were brokered among migrants that contributed to migrants’ prosperity in Ouagadougou. For example, while one migrant reported that he came by his plot of land through a personal connection who owned land near a small reservoir outside of Ouagadougou, he says that migrants often work together and that this cooperation can be facilitated through mobile phones,

You can call someone up and say, ‘Hey! Bring me some organic manure…two carts, three carts full. You can call to see if there are seeds available. ‘Bring me two sacks of seeds!’ You can also call to say that you need seeds, but you don’t have money. ‘I need two bags of seeds, but I don’t have any money. Bring them to me, and I can pay you back.’ All of that helps (Participant U, male, Boulmiougou interviews).

Where exchanges with home communities were characterized by requests for gifts, exchanges between migrants were characterized by informational exchanges, with the expectation that support networks could be used to provide a safety net, to buffer against lean harvests, and to bolster mutual fiscal viability.

Alongside connecting with employment opportunities and connecting with other migrants to form collectives to provide insurance against lean economic conditions, migrants also reported using their phones to engage in commerce. This was particularly true in the case of our migrant farmers who were growing tomatoes and strawberries near a local reservoir (Barrage de Boulmiougou). Mobile phones could reduce costs and ensure timely harvest. For example, the harvest of strawberries was often coordinated with the women who would eventually sell them in the market, according to respondents.

Telephones are useful. With strawberries you have to call around. ‘Hello! You coming? Should I start picking? Yes! We need to start picking! Or no—leave them, and I’ll come by tomorrow. (Participant V, male, Boulmiougou interviews)

And not only for ensuring timely harvest, mobile phones also enabled the transaction of business. The Barrage de Boulmiougou is located on the outside of the city and transportation was a considerable cost. Having access to mobile phone technology enabled growers to converse with potential sellers from as far away as the Ivory Coast. According to one participant,

Yes, phones are helpful. It’s like I told you, you can stay where you are and just call your clients directly. … We can even talk prices so that when he comes he just gives you the money and takes the product instead of wasting time in negotiations. I even sell to someone in the Ivory Coast. I can talk to him about prices, make the transaction, exchange the money, and send the product without ever seeing him. (Participant W, male, Boulmiougou interviews)

As a result, mobile phones have the potential to increase a grower’s potential market, to reduce the cost of transacting business, and to ensure the quality of the product.

In addition to reducing the cost of business, migrants have found ways in which to use mobile phones to contribute to the expansion of their business opportunities and to communicate their skill as growers. According to one respondent, strawberries are quite fragile and demand an in-depth understanding of agriculture. Thus, to demonstrate that he is capable of producing a high quality product, he carries a photo of his strawberries on his phone to demonstrate the quality of his product for potential investors. He says,

I took this picture to show that I know how to grow strawberries. I can show someone who may want to invest in me or my product. So it’s an opportunity. If I didn’t have a picture, they could take me for a liar. (Participant X, male, Boulmiougou interviews)

Given that many gardeners near the Barrage de Boulmiougou rented the land they use to garden, it may be important for landowners to know that they are renting to capable farmers. Using mobile telephone technology, Participant X is able to demonstrate that he is not lying about his abilities as a skilled gardener.

None of the women interviewed worked in agriculture or masonry. As a result, the ways in which women exchanged resources and found employment were unlike those of men. None of the women came unaccompanied to Ouagadougou. Two of the women came with spouses or partners, and our three single women came to stay with relatives who were already living in the city. Because of these existing relationships in the city, it may be that women had the financial stability and social relationships that enabled them to secure initial employment, before attempting to find better paying or more skilled labor. Nevertheless, our results indicated that few female migrants actually used their mobile phones to connect with economic opportunity and were able to capitalize on mobile phone technology to ease their financial situations. According to one participant, female migrants exchange phone numbers with men they met in the city. According to one participant,

There are advantages [to the mobile phone]. Sometimes they’re how we get to eat these days…or at least a beer plus 5.000 francs. (Participant S, female, focus group 4)

This participant refers here to calling interested men and offering to have sex in exchange for an evening out and 5,000 francs.

### Mobile phones as a catalyst and facilitator of domestic migration

Where evidence from transnational migration suggests that communication technologies can serve to catalyze chain migrations by providing information on host communities and creating realistic expectations of the migration experience, the role of mobile phones in domestic migration appears to be notably different. Our evidence suggests that mobile phones played a more limited role in motivating migration, and that migration was primarily motivated by cosmopolitan migrants returning to the village having made their fortune in the city. According to one migrant,

You see, we come to Ouagadougou to make money. When you’re at home in the village with your parents and your friends come back with money, they seem transformed. That’s what pushes you to come to Ouaga. (Participant K, male, focus group3)

The effect can be even more pronounced when migrants observe stark differences between their own situation and that of villagers their own age who left for Ouagadougou to make their fortunes. Participant K continues,

When you see people from your generation who leave for the city and who make money, when your own situation—and the situation of everyone else who stayed—fails to change, you also want to get to the city.

Where previous research has suggested that the transfer of information facilitated by communication technology may facilitate migration and provide a more realistic image of migration, the process of chain migration proposed by Perkins and Neumayer [26] may not hold in the case of domestic migration where ties to home communities are not completely severed and migrants have the option to return home. Rather, the maintenance of face-to-face ties in the community available to more successful migrants may create a selection effect whereby only successful migrants are willing and able to return to the village and would-be migrants get a skewed impression of the opportunity available in the city.

In addition to the limited role played by mobile phones in catalyzing domestic migrations or influencing expectations of the migration experience, we also found limited evidence that technology served to facilitate migration and integration into the city. Individuals’ initial migration experiences were primarily influenced by the degree to which they were able to set up face-to-face networks of support upon arrival. Most migrants’ stories include spending time in cafes and bars, befriending locals by buying them drinks in the hopes of being invited into someone’s home or being connected with work opportunities. Absent the finances to purchase drinks for locals, some unlucky migrants simply found themselves homeless.

Last time I made an appointment to talk to someone about a possible job, we agreed to meet up at a bar. And what are you going to do if you don’t have money to buy that person a beer. The bartender is not going to let you stay! (Participant J, male, focus group 2)

One respondent reported that he had migrated in from the village but was only able to find work running flowers from a roadside flower stand outside of a hospital to the cars that pulled up to purchase flowers. Wages were poor, so without social connections, he would spend his days working at the roadside flower stand and his nights sleeping outside the flower stand. In another instance, one man took a bus into Ouagadougou, but was dropped off several miles outside the city. He was forced to spend all of his money covering the remaining distance between where he’d been dropped off and the city limits, and arrived penniless, only able to secure employment washing dishes for a woman who had a roadside restaurant serving rice. Paid only 50 Central African Francs per day, the man was forced to find shelter under a bridge.

### Drawbacks of mobile phones and negative effects on migration

As early evidence is starting to emerge on the drawbacks of ICT-mediated exchange and migration, our evidence revealed that continuous access to communication with origin communities was not always welcome and could exacerbate pressures on migrants’ stemming from financial and social obligations. According to one participant,

Whenever there is a problem in the village, they always call the person who migrated to the city on the phone so that they can intervene. When there’s a death of a village elder, they call us and tell us to come home. But you need gas money to get there. But then if you don’t come, they criticize you, ‘Did you hear about this guy? He died and his kid didn’t even come home.’ (Participant K, male, focus group 3)

According to the migrants in our sample, despite continued communication between migrants and their home communities, there is a sense that villagers fail to understand the pressures and the economic constraints faced by migrants. Migrants are under pressure to provide support to their families and communities back home, but often lack the financial stability to be able to attend to those obligations. It can become so difficult for migrants that they end up using mobile phones to lie about their whereabouts. According to M,

Even I stayed here for a full year before I went home. It got to the point where they called me to ask when I was going to come home. I ended up telling them I was in Kongoussi (around 110 km north of Ouagadougou). (Participant F, male, focus group 2)

Along with continued obligations to members of the home community, there was a sense that mobile phones promoted secrecy, dishonesty, and the breakdown of family relationships. Parents could not be sure what their children were doing and had no way of monitoring their children’s social relationships. Through use of mobile phones, relationships found additional grounds for jealousy and suspicion. When talking about the ways in which mobile phones could make life more difficult, one participant reports,

It’s the same with women [as it is with men]. You can’t tell her not to answer the phone, but if it’s a man, she’ll answer and you’ll complain. Then she’ll say, ‘It’s my uncle’—but then if you get into a fight about it, it’s because you’re a jealous partner. (Participant A, male, focus group 1)

The theme of infidelity came up quite often among the men in the focus group interviews, but the tone was decidedly different based on whether it was the man or the woman who was engaging in the behavior. When the woman was having an affair, the mobile phone technology could destroy a family by facilitating clandestine interactions between men and women. However, in instances where men reported their own affairs, mobile phones “destroy families” by exposing and providing evidence to a man’s wife that he was having an affair.

Finally, migrants reported that mobile phones enabled migrants to disregard basic social norms. For example, one migrant discusses an occasion where he began flirting with a woman from his village at a party. Over the course of the evening, he promised to marry the woman despite his poor financial situation in the city. On several occasions, the respondent reported that the woman’s family would try to call and each time the respondent would avoid the call by saying he was out of town, hoping that the obligation would ultimately resolve itself. The technique proved effective for several months, until the family showed up in Ouagadougou to wait for the respondent at his apartment. He was so embarrassed about the state of the apartment, which had nothing—not even a mattress—that he just stayed away until they went home, taking his would-be bride back to the origin community

## Discussion

If migration is to be viewed as a livelihood diversification strategy, it should facilitate greater access to resources and information, expand and proliferate social networks, and alleviate financial obligations among communities of origin. Proponents of mobile phone technology to aid in economic and health development suggest that mobile phones should facilitate many of these elements of livelihood diversification [59,60], yet research on the role of mobile phones in technology have been equivocal. This study examines why and how by examining the role of mobile telephones in the migration process. Results indicate that communication technologies promote the sharing of information and resources; however, they fail to encourage the type of information sharing that can promote financial prosperity in the community of origin. In addition, by promoting pestering for financial resources and exchanges of resources ***from home communities to migrants***, communication technologies have the potential to undermine social ties and increase financial burdens on home communities. Nevertheless, there are ancillary benefits to communication technologies that have heretofore been under-acknowledged in migration studies.

Our study was motivated by the potential for mobile phone technology to facilitate the transmission of resources and information across geographic distances. In theory, the advent and widespread adoption of mobile phones should provide rural farmers and pastoralists with direct access to market and price information, while money transfer services like Airtel Money should provide quick a quick and convenient way for migrants to provide remittances to their home communities. Nevertheless, our data showed limited evidence that mobile phones promoted economic gain in migrants’ home communities: we show little to no evidence of either information or resource transfers from migrants to home communities via mobile phone. Rather than a fault of imagination, this failure to capitalize on mobile technology for the benefit of the home communities may be the result of a longstanding culturally valued tradition of what De Bruijn and Brinkman [61] refer to as “bush-falling,” or returning to home communities with gifts for friends and family members.

Conversely, the communication of need and care-taking–from home community to migrant, and from migrant to home community–was facilitated by mobile phone use. Women who had been separated from children were able to call and check in frequently, to inquire about their children’s health, and to discuss any financial needs of the child or the family with whom the child had been entrusted. Nevertheless, according to our respondents, this frequent communication could quickly take on a ***pestering*** quality [24], as home communities made relentless demands of the migrant for both financial resources and social obligations: including making good on promises of marriage, attending funerals, and mediating conflict between members of the community. According to migrants, this ***pestering*** constituted an unwelcome monitoring of migrants lives in the city and could encourage migrants to capitalize on mobile technology to lie about their whereabouts, and to avoid communication with members of their home communities. So, while mobile phones permit pestering and may add to migrants’ stress, they may advantage migrants at the expense of home communities by enabling them to restrict communication, lie regarding whereabouts, and ultimately skirt financial and social obligations.

There are other ways in which mobile phones advantage migrants, often at the expense of home communities. According to our research, while research on the utility of mobile phones in migration has typically focused on the transfer of resources ***from the migrant to their home community***, mobile phones also permit communication of need ***by the migrant to their home community***. As a livelihood diversification strategy, the migration of a young person should alleviate some financial strain within the household. However, our results show that migrants are able to communicate their own financial needs to friends and family back home, in order to access financial resources, creating additional financial burden on families at home. In addition, where mobile phones did prove facilitative in forging new relationships, they were often made between migrants, themselves. Our evidence revealed compelling evidence that migrants leveraged common experience, shared knowledge and brokered resources amongst themselves, but rarely did that brokering result in benefits and expanded social networks for the sending community.

### Directions for future research

Qualitative research relies on rich data from a restricted and often non-representative sample to identify the mechanisms and motivations that may underlie observed relationships, such as introduction of cell phones into the migration process. Our study highlights myriad pathways through which mobile telephony can both facilitate and hinder migration, and how those relationships can differ. Our results highlight how cell phones can facilitate migration by shoring up family and community relationships, allowing migrants to access important resource, information and emotional support, and allowing migrants to attend to important community obligations in absentia. Our results also echoed other research suggesting that cell phones may be a burden to migrants by allowing for constant pestering for resources with limited exchange of empowering information. Results also included unanticipated findings related to use of cell phones to facilitate the rapid establishment of social and professional networks between migrants. Each of these pathways represents a potential direction for future quantitative research to test the generalizability of the pathways highlighted in this study. Notably, does continued communication with children and family via cell phones serve to enable migration among women or does it increase women’s unpaid labor? Does early cell phone investment among masons and farmers result in greater long-term economic opportunity?

## Conclusion

Migration has long been considered as an effective strategy for livelihood diversification: alleviating financial constraints while promoting increased access to both resources and information. In theory, mobile telephones should facilitate livelihood diversification by enabling the rapid transmission of information and resources, making migration even more valuable and fostering even more development in home communities. Our results show that mobile phones may actually dampen the benefits of migration on sending communities, advancing the development of migrants’ own social networks, often at the expense of home communities.
